# SIRT2 gene has a classic SRE element, is a downstream target of serum response factor and is likely activated during serum stimulation

**DOI:** 10.1371/journal.pone.0190011

**Published:** 2017-12-21

**Authors:** Xiaomin Zhang, Gohar Azhar, Jeanne Y. Wei

**Affiliations:** Donald W. Reynolds Institute on Aging, University of Arkansas for Medical Sciences, Little Rock, Arkansas, United States of America; Hokkaido Daigaku, JAPAN

## Abstract

The sirtuin proteins are an evolutionarily conserved family of NAD^+^-dependent deacetylases that regulate various cellular functions. Among the seven sirtuins, SIRT2 is predominantly found in the cytoplasm, and is present in a wide range of tissues. Recent studies indicate that SIRT2 plays an important role in metabolic homeostasis. Several studies indicate that SIRT2 is upregulated under serum deprivation conditions. Since the serum response factor gene usually responds rapidly to serum deprivation and/or serum restoration following deprivation, we hypothesized that a common mechanism may serve to regulate both SIRT2 and SRF during serum stimulation. Using a bioinformatics approach, we searched the SRF binding motif in the SIRT2 gene, and found one classic CArG element (CCATAATAGG) in the SIRT2 gene promoter, which was bound to SRF in the electrophoretic mobility shift assay (EMSA). Serum deprivation induced SIRT2 expression, while SRF and the SRF binding protein, p49/STRAP, repressed SIRT2 gene expression. SIRT2 gene expression was also repressed by the Rho/SRF inhibitor, CCG-1423. These data demonstrate that the classic SRE element in the SIRT2 gene promoter region is functional and therefore, SIRT2 gene is a downstream target of the Rho/SRF signaling pathway. The increased expression of SRF that was observed in the aged heart may affect SIRT2 gene expression and contribute to altered metabolic status in senescence.

## Introduction

The sirtuin proteins are an evolutionarily conserved family of NAD^+^-dependent deacetylases that regulate various cellular functions [1]. Seven mammalian homologs have been identified (SIRT1–7) in mammals [2–5]. They share a conserved central deacetylase domain but have different N- and C- termini and display distinct subcellular localizations, suggesting potentially different biological functions [6]. For example, SIRT1 is mainly localized in the nucleus; SIRT2 is mainly in the cytoplasm; SIRT3, SIRT4, and SIRT5 are often in mitochondrion; and SIRT6 and SIRT7 are usually in the nucleus. Sirtuin enzyme activity is regulated by the intracellular NAD/NADH ratio, which may be influenced by metabolic status, including nutrient stress, caloric restriction and energy excess.

Among the seven sirtuins, SIRT2 is predominantly found in the cytoplasm, and is present in a wide range of tissues. Recent studies indicate that SIRT2 plays an important role in metabolic homeostasis [7–9]. Some studies indicate that SIRT2 is upregulated under serum deprivation conditions. Since the serum response factor gene usually posts a quick response to serum deprivation and/or serum restoration following deprivation, we hypothesized that a common mechanism may serve to regulate both SIRT2 and SRF during serum stimulation.

In a previous study, we identified 192 genes that had SRF binding motifs, the classic CArG or CArG-like elements [10]. Using a similar bioinformatics approach in this study, we searched the SRF binding motif in the SIRT2 gene, and found one classic CArG element (CCATAATAGG) in the SIRT2 gene promoter, which we found to be bound to SRF in the electrophoretic mobility shift assay (EMSA). Serum deprivation induced SIRT2 expression, while SRF and the SRF binding protein, p49/STRAP, repressed SIRT2 gene expression. Our findings suggest that SIRT2 is a classic SRF target gene and is regulated by the Rho/SRF signaling pathway in response to serum stimulation.

## Materials and methods

### Analysis of SRF binding sites: The classic CArG motifs in the gene promoter

The criteria for the classic CArG motif is a 10-bp element that has the sequence CC(A/T) _6_GG. Briefly, the mRNA sequences were obtained from the NCBI Reference Sequence Database (RefSeq) for human SIRT2 gene. The reference mRNA sequences was submitted to BLAST for comparison with the human genomic DNA sequence in the human genome database. After noting the orientation of the alignment, the appropriate 5-Kb genomic DNA sequence upstream from the transcription start point corresponding to the promoter region of the gene was isolated and analyzed using a web-based bioinformatics tool TESS at http://www.cbil.upenn.edu/cgi-bin/tess/tess. Because TESS results are model-based, the potential CArG sequence was verified by both using LALIGN (http://www.ch.embnet.org/software/LALIGN_form.html) and visual confirmation.

### Cell lines, genomic DNA and plasmid constructs

Human genomic DNA samples were purchased from Zyagen (San Diego, CA). C2C12 cells, HEK293 cells, Hela cells, HT29 cells and SW480 cells were obtained from ATCC (Manassas, VA). A 645 bp DNA fragment containing part of the human SIRT2 promoter (-4029 bp to -3384 bp upstream from SIRT2 transcription start site) was amplified with the forward primer 5’-ggcctcaagaatatttataggaatacagaa-3’ and the reverse primer 5’-aaaatagttatctggctccttacagaaaaa-3’, and then cloned into pGL3-basic luciferase vector. The pCDNA3-SRF, pcDNA3-p49/STRAP were generated by cloning SRF and p49/STRAP cDNAs into the pcDNA3 vector, respectively. The pcDNA3-Myocardin expression construct was a gift of Dr. Olson (University of Texas Southwestern Medical Center, Dallas, TX). All the plasmid constructs were verified by sequencing analysis.

### Electrophoretic mobility shift assay (EMSA)

Two double strand DNA oligo probes derived from human SIRT2 promoter were used in the EMSA assay, respectively. One DNA oligo probe containing a classic CArG sequence 5′- GGAAAACATGAT***CCATAATAGG***AACCATCAGT -3′, and the other oligo probe containing a CArG-like sequence 5’-CAAAAACTATACC*CAATAAAAGG*CAAAAAGGAGGG-3’ were synthesized and labeled with radioactive [γ-32P]-ATP with the method that was described previously [11]. In vitro Translation of SRF Protein: The cDNA corresponding to the full-length coding region of SRF was subcloned into plasmid pBluescript SK (-). The transcription of SRF genes was under the control of T7 promoter. The SRF protein was in vitro translated by using a TNT-coupled transcription/translation system (Promega) and was used for electrophoretic mobility shift assays (EMSAs). The cell lysate of C2C12 cells was also used for the EMSA. The oligonucleotides were labeled with [γ-32P]-ATP using T4 polynucleotide kinase. Binding reaction mixtures were incubated at room temperature for 20 min and contained 0.5 ng of DNA probe and 5 ug of protein in the binding buffer with 10 mM Tris (pH 7.5), 50 mM NaCl, 1 mM dithiothreitol, 1 mM EDTA, 5% glycerol, and 1 ug of poly(dI-dC) to inhibit nonspecific binding of labeled probe to the in-vitro translation system or cell lysate. DNA-protein complexes were resolved by electrophoresis through 4% native polyacrylamide gels containing 50 mM Tris, 45 mM boric acid, 0.5 mM EDTA. The gels were subsequently dried and exposed to Kodak X-Omat film.

### Cell culture and transfection assays

Cell culture reagents were obtained from Fisher Scientific (Richardson, TX). Rho/SRF inhibitor CCG-1423 (EMD Millipore) was dissolved in DMSO, and 10 uM final concentration of CCG-1423 was used to treat the cells. When CCG-1423 solution was used for cell treatment, equal volume of DMSO was used as control.

The Hela cells were maintained in DMEM containing 10% FBS. For serum deprivation treatment, the cells were washed with PBS twice and then incubated in DMEM containing 0.1% serum for 3, 6, 24, and 48 hours as indicated.

For serum deprivation and restoration treatment, the cells were first subjected to serum deprivation for 48 hours and then were cultured in DMEM containing 10% FBS for additional 6, 18, 24, and 48 hours.

The plasmids for the transfection assay were pGL3-SIRT2 promoter-luciferase, pcDNA3 empty vector, pcDNA3-wtSRF, pcNDA3-p49/STRAP and pcDNA3-myocardin. In the transfection assays, Hela cells were transfected with complexes of 200 ng of reporter plasmid (pGL3-SIRT2-luciferase) and 200 ng of pcDNA3-wtSRF plasmid, pcNDA3-p49/STRAP and pcDNA3-myocardin, respectively. The plasmid pRL-CMV renilla luciferase control reporter vector (Promega; 10 ng/well) was transfected at the same time as an internal control. Transient transfections were carried out with a LipofectAMINE 2000 transfection kit (Life Technologies, Inc.). Approximately 4 h after the transfection was initiated, Hela cells were placed in DMEM containing 10% newborn bovine serum and incubated at 37°C overnight. The cells were then washed with 2 ml of serum-free DMEM and cultured in DMEM containing 0.1% serum for another 48 h. The DMEM containing 0.1% newborn bovine serum was then replaced by DMEM containing 10% newborn bovine serum, and the cells were cultured for an additional 3 hours. Firefly luciferase activity was measured as relative light units using a dual luciferase reporter assay system (Promega). The number of relative light units from individual transfection experiments was normalized by measurement of Renilla luciferase activity expressed from a cytomegalovirus promoter-driven vector in the same samples. Individual transfection experiments were carried out in triplicate, and the results were reported as mean firefly luciferase/Renilla luciferase activity (mean± S.D.) from at least three separate experiments.

### Measurement of gene expression

All RNA samples were isolated from the cultured cells using miRNeasy Mini Kit (Qiagen) and RNase-free DNase I digestion according to the manufacture’s instruction manual [12]. The reverse transcription and RT-PCR reagents were purchased from Applied Biosystems. The PCR amplification of SIRT2 gene was performed in a StepOnePlus Real-Time PCR System (Applied Biosystems) using the forward primer 5’-gccctttaccaacatggctg-3’, and the reverse primer 5’-gcgccccagttaggtaagaa-3’. Relative expression values were obtained by normalizing CT values of the mRNA genes in comparison with CT values of the endogenous control (5S RNA) using the CT method [12].

### Statistical analysis

Data are given as mean values ± SD, with n denoting the number of experiments unless otherwise indicated. A two-tailed T-test was used to determine the differences between the two groups. Bonferroni correction was applied to multiple comparisons. A P value of <0.05 was considered to be statistically significant.

## Results

### The SIRT2 gene locus

**Fig 1:**
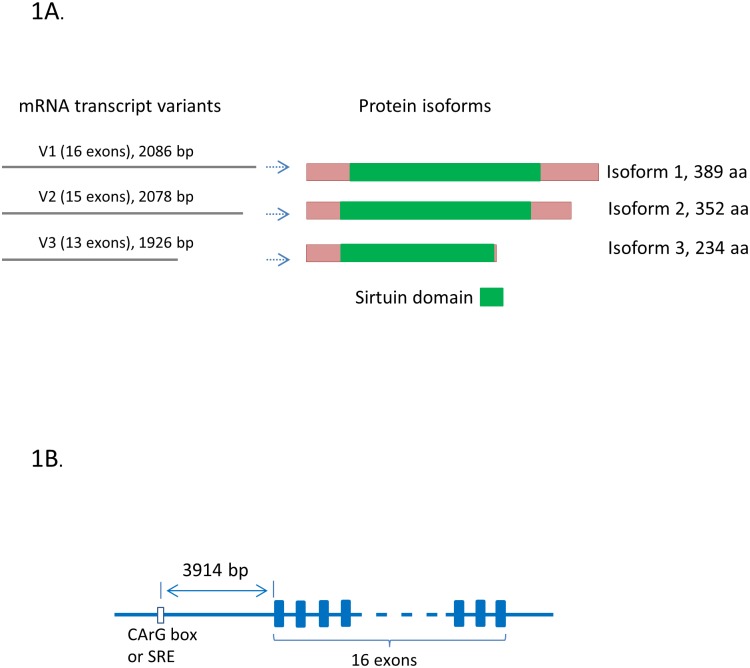
Human SIRT2 gene isoforms and the position of CArG sequence. **1A.** The SIRT2 gene has three mRNA transcript variants, which may translate to three protein isoforms. The main functional domain is Sirtuin domain (In green color). **1B.** The classic “serum response element” (SRE or CArG) sequence is 3914 bp upstream of transcription start site.

The human SIRT2 gene locus is located on chromosome 19. It is on the reverse strand of the chromosome 19 between 38836378–38933020. Its neighboring genes are the coiled-coil glutamate rich protein 2 (CCER2) gene and the Ras and Rab interactor like (RINL) gene. The SIRT2 gene has 16 exons, which transcribes into three mRNA transcript variants, and may translate to three protein isoforms (Fig 1A).

### A classic serum response element (SRE)/CArG-box exists in the promoter of SIRT2 gene

**Fig 2:**
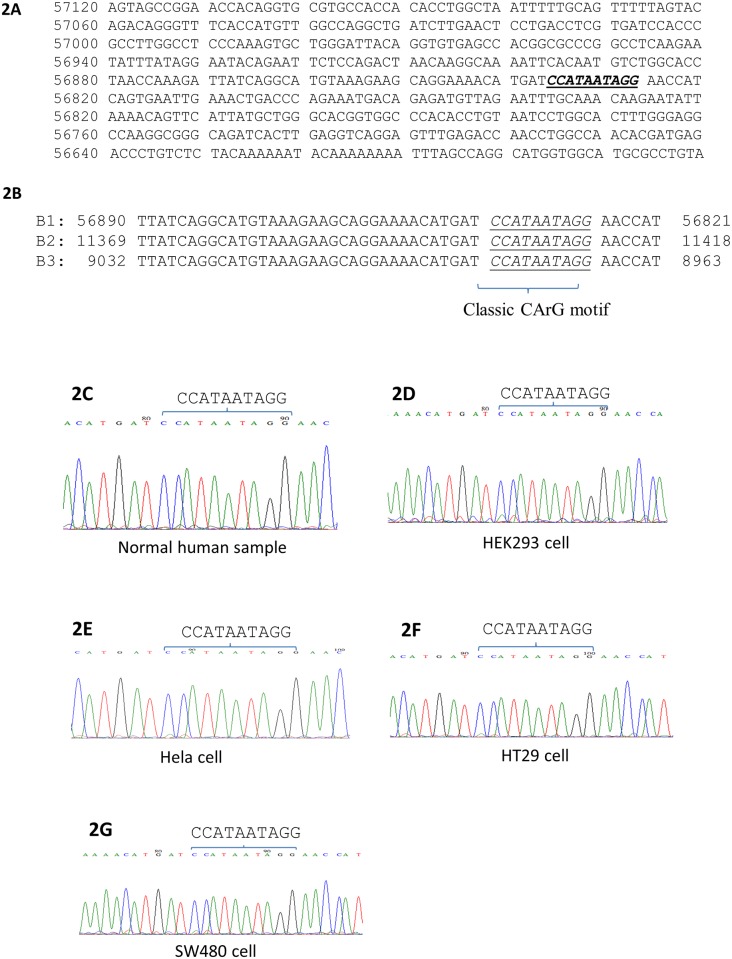
The classic SRE/CArG sequence in human SIRT2 gene promoter region. **2A.** Part of the DNA sequence from chromosome 19 clone CTC-360G5 (accession no. AC011455), showing a classic SRE sequence CCATAATAGG. **2B**. Alignment of three DNA sequences that have been deposited in the GenBank database. B1: Homo sapiens chromosome 19 clone CTC-360G5, complete sequence (AC011455). B2: Homo sapiens BAC clone CH17-66M11 from chromosome 19 (AC256309). B3: Homo sapiens RefSeqGene on chromosome 19 (NG_029624). **2C-2G**. Sequencing chromatograms of five DNA samples confirm the existence of a classic CArG motif “CCATAATAGG” sequence in the human SIRT2 gene promoter region. These DNA samples are from normal human donor (2C), HEK293 cells (2D), Hela cells (2E), HT29 cells (2F) and SW480 cells (2G).

In another study, we found classic CArG-box and CArG-like box sequences in a number of genes that had not been previously reported as SRF-target genes [10]. Using the similar bioinformatics approach in the present study, we analyzed the promoter sequence of SIRT2 gene, we found a classic CArG-box sequence “CCATAATAGG” in the promoter of SIRT2 gene, which is 3914 bp upstream of SIRT2 mRNA transcript (Figs 1B and 2A, accession number AC011455). Further search found two additional genomic DNA sequences (accession numbers AC256309 and NG_029624) deposited in the Genbank which cover the SIRT2 gene promoter region. As shown in Fig 2B, all three genomic DNA sequences revealed that the same CArG motif sequence existed in the SIRT2 gene promoter region.

To verify the bioinformatics findings, we sequenced five DNA samples, which all confirmed the same CArG-box sequence. The classic CArG-box sequence “CCATAATAGG” was found to be present in the DNA sample from a healthy human individual (Fig 2C), in HEK293 cells (Fig 2D), in Hela cells (Fig 2E), in HT29 cells (Fig 2F) and in SW480 cells (Fig 2G).

### Serum response factor protein binds to the CArG-box of SIRT2 gene promoter

**Fig 3:**
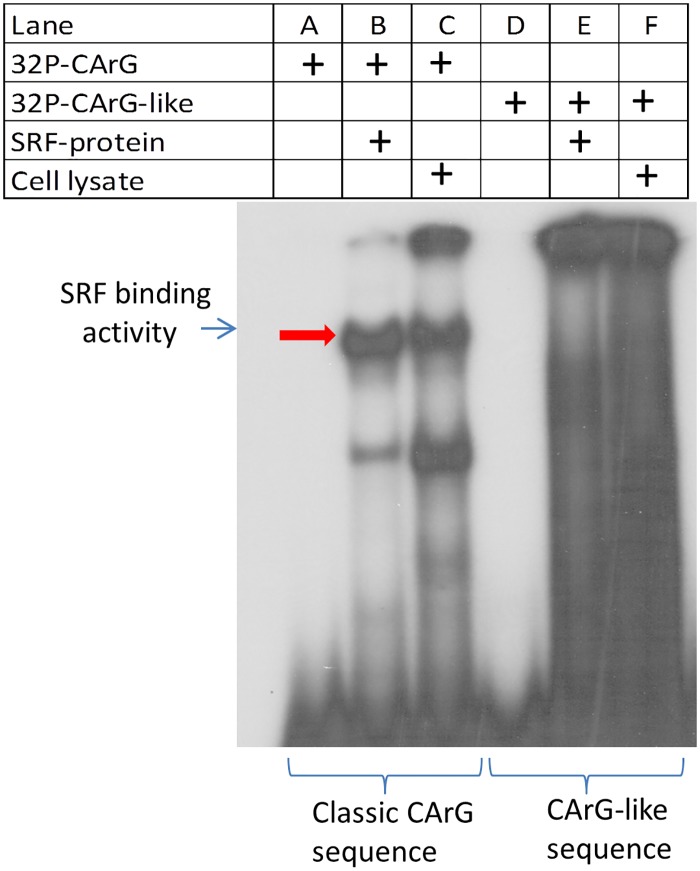
Electrophoretic mobility shift assay (EMSA) confirms that SRF protein binds to SIRT2 promoter sequence containing the classic CArG box (CCATAATAGG). To ensure that it is the SRF protein that binds to hSIRT2 CArG sequence, in-vitro synthesized SRF protein was used in the binding assay. EMSA indicates that purely synthesized SRF protein binds to classic CArG box (32P-CArG, lane b), but SRF does not bind to CArG-like sequence (CAATAAAAGG)(lane E) in which a “C” is substituted with a “A”. The arrow indicates the binding activity of full-length of SRF protein to the classic CArG sequence, which is at the similar position as what was observed in our previous experiments [33].

SRF regulates its target genes through binding to the CArG box sequence [13]. To test whether SRF could bind to the SIRT2 gene promoter sequence, the electrophoretic mobility shift assay (EMSA) was performed. A DNA oligo of SIRT2 gene promoter containing the classic CArG-box sequence “CCATAATAGG” was used as a probe. In addition, a DNA oligo containing a CArG-like sequence “C*A*ATAAAAGG”, in which a “C” was substitute by A, was also used as a probe for comparison. To demonstrate that it is the SRF protein that binds to the CArG-box “CCATAATAGG” probe, the pure SRF protein that was obtained with in-vitro translation system using SRF cDNA as template was used in the binding assay; cell lysate from the C2C12 muscle cell line was used for comparison. As shown in Fig 3 in lane B, in-vitro synthesized pure SRF protein was firmly bound to the CArG-box oligonucleotides of hSIRT2 gene promoter during electrophoresis; C2C12 cell lysate with native SRF also bound to CArG-box oligonucleotides in lane C. However, pure SRF protein did not bind to CArG-like oligo probe (lane E in Fig 3). These data indicates that SRF binds to the classic CArG-box of SIRT2 promoter, therefore, SIRT2 gene is a SRF-target gene.

### Effect of SRF and SRF-binding proteins on SIRT2 promoter activity

**Fig 4:**
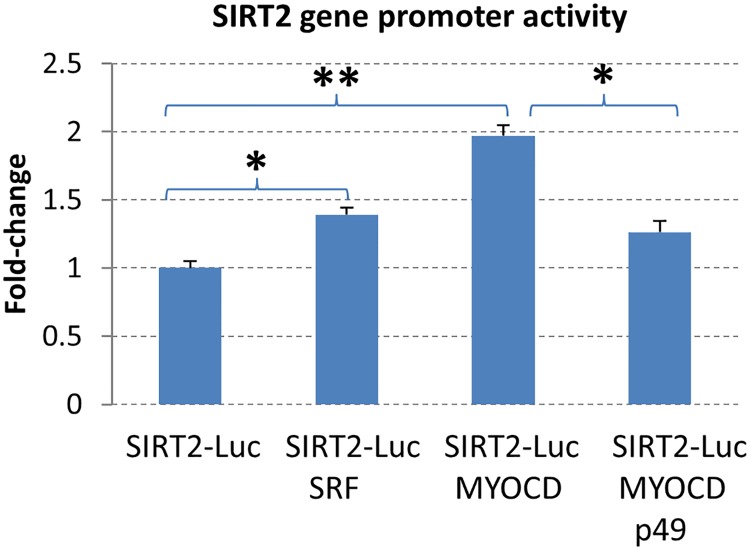
SRF and SRF-binding proteins regulate SIRT2 gene promoter activity. SRF upregulated the SIRT2 gene promoter activity by 1.39 fold + 0.051 (p< 0.05*, n = 3); SRF and myocardin synergistically activated SIRT2 gene promoter activity by 1.97 fold + 0.079 (p< 0.01**, n = 3); while p49/STRAP repressed the SIRT2 promoter activity (1.26 fold + 0.053, p< 0.05*, n = 3) that was induced by myocardin.

SRF regulates genes by binding to the CArG-box sequence in the gene promoter, and interacting with SRF-binding proteins. These interactions likely modulate SRF function and may also enable SRF to mediate tissue-specific regulation of its target genes at different physiological and pathological conditions. To test the effect of SRF and its binding proteins on SIRT2 gene expression in vitro, a luciferase expression construct containing a classic CArG sequence of the SIRT2 gene promoter was generated. The SIRT2-luciferase plasmid was then co-transfected into Hela cells with SRF, p49/STRAP and myocardin, respectively. After serum deprivation and serum restoration (detailed in Method section), the cells were harvested and the luciferase activity was measured. SIRT2-Luciferase plasmid was used as control. As shown in Fig 4, SRF upregulated the SIRT2 gene promoter activity by 1.39 fold ± 0.051 (p< 0.05*, n = 3); SRF and myocardin synergistically activated SIRT2 gene promoter activity by 1.97 fold ± 0.079 (p< 0.01**, n = 3); while p49/STRAP repressed the SIRT2 promoter activity (1.26 fold ± 0.053, p< 0.05, n = 3) that was induced by SRF and myocardin.

### Effect of serum deprivation and restoration on SIRT2 gene expression

**Fig 5:**
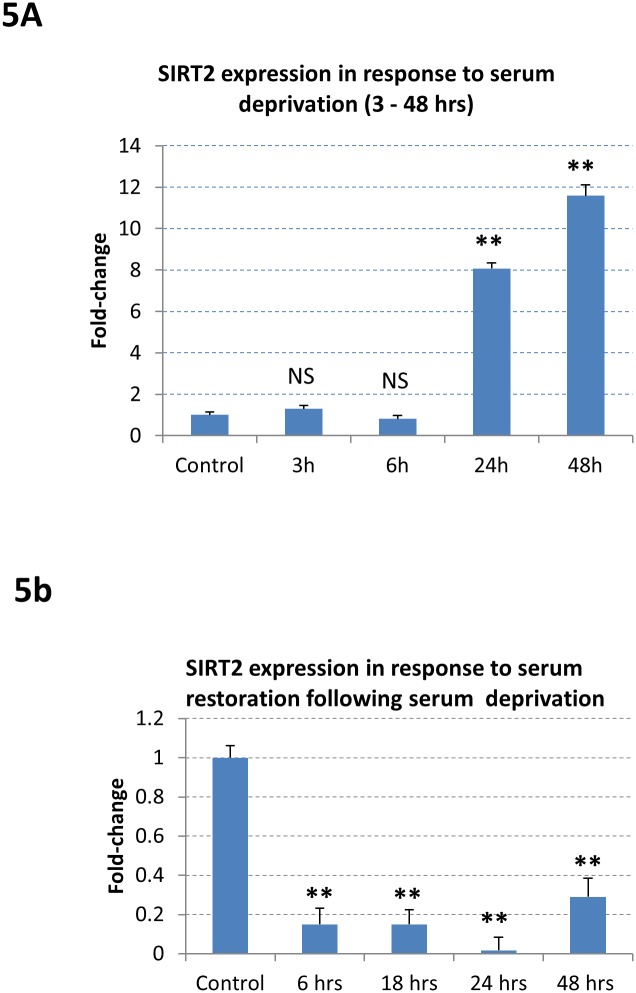
The response of SIRT2 mRNA expression to serum deprivation and serum restoration following serum deprivation. **5A.** The Hela cells were initially maintained in DMEM medium (glucose concentration: 4.5 g/L) containing 10% FBS. After washing with PBS twice, the cells were subjected to serum deprivation stress with DMEM containing 0.1% FBS for 3 hours, 6 hours, 24 hours and 48 hours, respectively. SIRT2 expression is increased from 24h to 48h after serum deprivation. **5B.** For the treatment of serum restoration following serum starvation, the cells were first cultured in DMEM containing 0.1% serum for 48 hours, then were cultured in DMEM containing 10% serum for additional 6, 18, 24, and 48 hours, respectively. SIRT2 expression was decreased after 6 hours serum restoration (0.15+0.08, p<0.01**, n = 3), 18 hours serum restoration (0.15+0.07, p<0.01**, n = 3), 24 hours serum restoration (0.018+0.06, p<0.01**, n = 3) and 48 hours serum restoration (0.29+0.09, p<0.01**, n = 3).

The SRF protein and serum response element (or CArG-box) were initially discovered in cells that were manipulated with serum starvation and serum restoration, where a SRF-target gene (c-fos) was activated after serum starvation followed by serum restoration [13,14]. In the present study, the Hela cells were treated with either serum starvation only, or with serum restoration following serum starvation. For serum deprivation treatment, the cells were cultured in DMEM containing 0.1% serum for 3, 6, 24, and 48 hours. As shown in Fig 5A, SIRT2 gene expression did not change significantly after 3 hour serum deprivation (1.2±0.15, p >0.05, NS, n = 3) and 6 hour serum deprivation (0.8±0.17, p >0.05 NS, n = 3) compared to control (1±0.14, n = 3). However, SIRT2 gene expression was increased significantly after 24 hour serum deprivation (8.1±0.28, p <0.01**, n = 3) and after 48 hour serum deprivation (11.6±0.51, p <0.01**. n = 3).

For the treatment of serum restoration following serum starvation, the cells were first cultured in DMEM containing 0.1% serum for 48 hours, then were cultured in DMEM containing 10% serum for additional 6, 18, 24, and 48 hours, respectively. As shown in Fig 5B, SIRT2 expression was decreased after 6 hours serum restoration (0.15±0.08, p<0.01**, n = 3), 18 hours serum restoration (0.15±0.07, p<0.01**, n = 3), 24 hours serum restoration (0.018±0.06, p<0.01**, n = 3) and 48 hours serum restoration (0.29±0.09, p<0.01**, n = 3). These data indicate that serum concentration affects SIRT2 gene expression, as is the case with a number of other SRF-target genes [15].

### SIRT2 gene is a downstream target of Rho/SRF signaling pathway

**Fig 6:**
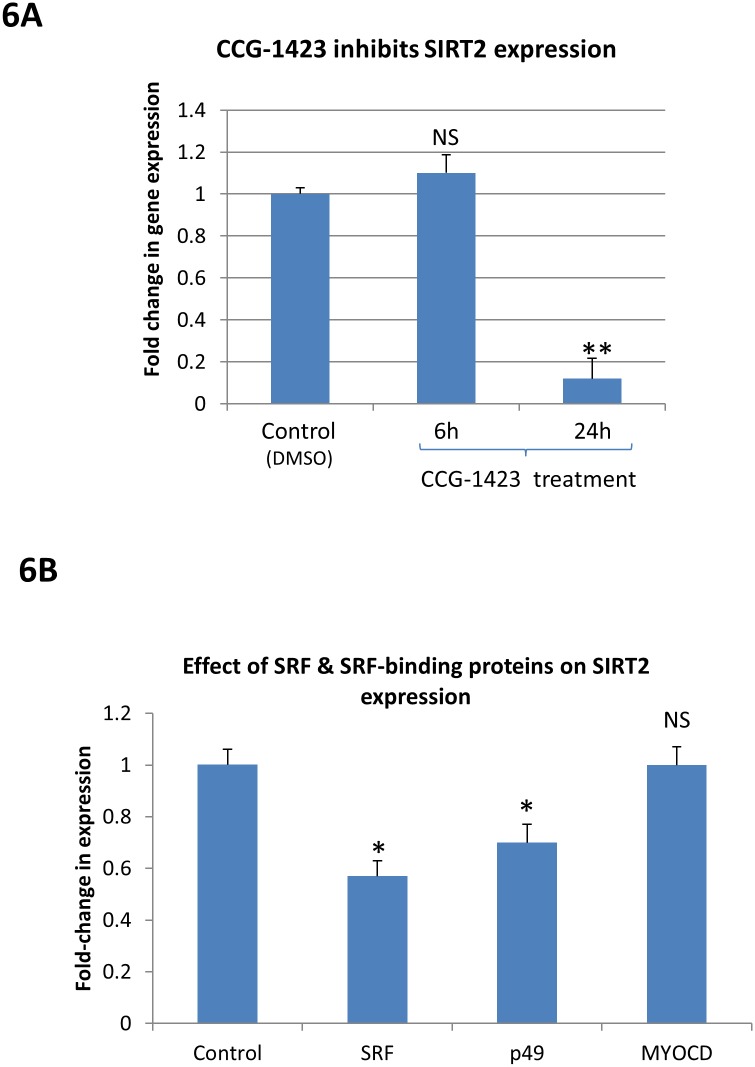
SIRT2 gene is regulated by Rho/SRF signaling pathway and SRF cofactors. **6A.** SIRT2 expression in response to serum deprivation did not change after 6 hours of CCG-1423 treatment (1.1+0.08, P>0.05 NS, n = 3), but was significantly inhibited after 24 hours of CCG-1423 treatment (0.2+0.09, p< 0.01**, n = 3), indicating that the Rho/SRF signaling pathway regulates SIRT2 gene expression. **6B.** The SIRT2 expression was repressed by both SRF (0.57+0.06, p<0.05*, n = 3) and p49 (0.7+0.07, p<0.05*, n = 3) under normal culture condition without stress, suggesting that SRF and its cofactors regulate expression of the SIRT2 gene.

To examine whether SIRT2 gene expression may be regulated by the Rho/SRF signaling pathway, the Rho/SRF inhibitor CCG-1423 was used in the serum deprivation test. As shown in Fig 6A, SIRT2 mRNA expression in response to serum deprivation did not change after 6 hours of CCG-1423 treatment (1.1±0.08, P>0.05 NS, n = 3), but was significantly inhibited after 24 hours of CCG-1423 treatment (0.2±0.09, p< 0.01**, n = 3), indicating that the Rho/SRF signaling pathway regulates SIRT2 gene expression.

The expression of SIRT2 mRNA was also measured in cultured Hela cells transfected with SRF, p49 and myocardin plasmid constructs. As shown in Fig 6B, the SIRT2 expression was repressed by both SRF (0.57±0.06, p<0.05*, n = 3) and p49 (0.7+0.07, p<0.05*, n = 3) under normal culture condition without stress, suggesting that SRF and its cofactors regulate expression of the SIRT2 gene.

## Discussion

The present study has several major findings. A classic SRE cognate response element (CCATAATAGG) was found to exist in the SIRT2 gene promoter, which was observed to be tightly bound to SRF protein in the EMSA assay. Similar to the other SRF target genes that have a SRE cognate response element, including the c-fos gene, the SIRT2 gene responded strongly to both serum deprivation alone as well as to serum restoration following a period of serum deprivation. SIRT2 gene expression was found to be repressed by the Rho/SRF inhibitor, CCG-1423[16]. In addition, SIRT2 gene was observed to be regulated by SRF and two of its cofactors, p49 and myocardin. These data demonstrate that the classic SRE element in the SIRT2 gene promoter region is functional and therefore, SIRT2 gene is a downstream target of the Rho/SRF signaling pathway.

It is well appreciated that the mammalian adult heart undergoes a number of changes with advancing age. Serum response factor (SRF) is an important transcription factor that regulates cardiac and skeletal muscle genes during development, maturation and adult aging [17,18]. SRF regulates its target genes by binding to serum response elements (SREs), which contain a consensus CC(A/T)_6_GG (CArG) motif. This cognate binding site of SRF is found in the promoter region of certain immediate-early genes and a number of muscle specific genes. Recent studies have revealed that the CArG motif exists in many genes with various functions [19,20]. These findings indicate that SRF may play a role in the regulation of a wide range of cellular processes, including cell proliferation, nutrient stress and cell survival [10].

The SRF protein and the serum response element were initially discovered in cells undergoing serum deprivation followed by serum restoration, which mimics the clinical manifestation of blood vessel blockage and reopening that has been observed in patients [14]. SRF forms a dimer to bind to the serum response element (SRE) or CArG motif sequence. The SRE or CArG motif was initially found in the promoter region of immediate-early genes, including c-fos and c-jun. Later, the CArG motif has been found in the promoter of a number of cardiac, skeletal and smooth muscle genes, which include α-MHC, β-MHC, cardiac and skeletal actin [17,20]. Recent studies have revealed that the CArG motif exists and is functional, not only in immediate-early genes and cytoskeletal genes, but also in many other genes with various important functions [21]. For example, the study that was conducted by our group found 51 genes with classic SRF binding sites (CArG motif sequences) that had not been previously reported, including genes which are important in energy metabolism, xenobiotic metabolism, stress response, immune response and cytoskeleton [10].

The “CCATAATAGG” sequence in the SIRT2 gene promoter is a classic SRE, which allows the SRF dimer to firmly bind to the DNA sequence, whereupon various SRF-binding proteins may be recruited to form a protein complex, which in turn modulates the expression of the SIRT2 gene. The composition of the protein complex of SRF and SRF cofactors may vary among different tissues, cell types, developmental stages and also in response to various physiological and pathological conditions [22]. In the present study, SRF upregulated the SIRT2 gene promoter luciferase activity which contained a short piece of the SIRT2 gene promoter. In contrast, SRF protein repressed the SIRT2 mRNA expression in cultured cells. This difference in response suggests that although the promoter-luciferase constructs, which could be in either wild-type or mutant version, can be used to examine the SRF and its cofactors in the transfection assay, but the interpretation of its role in transcriptional regulation may differ. Indeed, the regulation of SIRT2 gene expression is far more complex, as the entire SIRT2 gene promoter is very long, and many other transcription factors and cofactors are likely to be involved in the regulation of the SIRT2 gene. This is the nuance of gene regulation that occurs under varying circumstances, from moment-to-moment, in different cell type and environmental conditions. Furthermore, gene transcription involves the assembly of RNA polymerase II with its multiple constituents at core promoters and its cell-type-specific activation by enhancers that can be located more distally [23]. Epigenetic changes are also influential here, and future studies will be of interest [24].

The SRF cofactors that may modulate SRF target gene expression include the ternary complex factors (TCFs), myocardin-related transcription factor (MRTF), p49/STRAP and other SRF-binding proteins [25–28]. The TCF family of SRF cofactors is regulated by ERK-MAP kinase pathway. For example, activation of ERK by 12-O-tetradecanoyl phorbol-13-acetate (TPA) stimulation was reported to induce a common pattern of histone modification at transcription start sites (TSSs) of SRF target genes [29]. The myocardin-related transcription factors (MRTFs) are regulated by Rho kinase signaling pathway [30].

Among SRF cofactors, the TCF cofactor, myocardin and MRTFs are known to bind to the SRF N-terminus, while p49/STRAP protein has been observed to bind to the SRF transcriptional activation domain [31]. It has been shown that the p49/STRAP protein is usually located within a protein complex containing the SRF and myocardin proteins. Therefore, p49/STRAP is able to modulate the transcriptional activation regulated by SRF [25,31].

**Fig 7:**
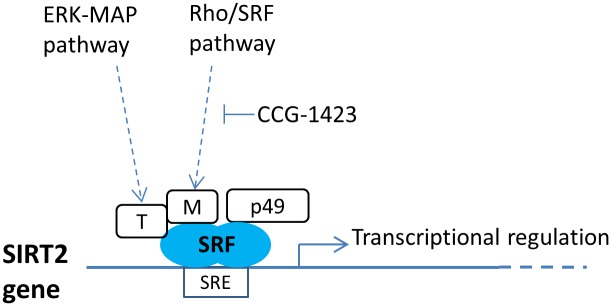
A proposed model of transcriptional regulation of SIRT2 gene by the Rho/SRF pathway, ERK-MAP pathway and other associated SRF cofactors, including p49/STRAP. This model is proposed based on the findings observed in the present study and data in the literature [25,26,29,32]. T, refers to TCF family of SRF cofactors. M, refers to myocardin-related transcription factor (MRTF) SRF cofactors.

Based on the findings observed in the present study and data in the literature [25,26,29,32], we hereby propose a model of transcriptional regulation of the SIRT2 gene via both the ERK-MAP and the Rho/SRF pathways and also the potential modulation by p49/STRAP protein (Fig 7). Future studies to elucidate their competing or synergistic interactions will be of interest.
